# Controlling the Carbon-Bio Interface via Glycan Functional Adlayers for Applications in Microbial Fuel Cell Bioanodes

**DOI:** 10.3390/molecules26164755

**Published:** 2021-08-06

**Authors:** Alessandro Iannaci, Adam Myles, Timothé Philippon, Frédéric Barrière, Eoin M. Scanlan, Paula E. Colavita

**Affiliations:** 1 School of Chemistry, CRANN and AMBER Research Centres, Trinity College Dublin, College Green, Dublin 2, Ireland; aleiannaci@libero.it (A.I.); amyles@tcd.ie (A.M.); 2 Institut des Sciences Chimiques de Rennes-UMR 6226, CNRS, Univ Rennes, F-35000 Rennes, France; timothe.philippon@univ-rennes1.fr

**Keywords:** aryldiazonium, functionalization, carbon, microbial fuel cells, bioanodes, electrocatalysis, bioelectrochemical systems

## Abstract

Surface modification of electrodes with glycans was investigated as a strategy for modulating the development of electrocatalytic biofilms for microbial fuel cell applications. Covalent attachment of phenyl-mannoside and phenyl-lactoside adlayers on graphite rod electrodes was achieved via electrochemically assisted grafting of aryldiazonium cations from solution. To test the effects of the specific bio-functionalities, modified and unmodified graphite rods were used as anodes in two-chamber microbial fuel cell devices. Devices were set up with wastewater as inoculum and acetate as nutrient and their performance, in terms of output potential (open circuit and 1 kΩ load) and peak power output, was monitored over two months. The presence of glycans was found to lead to significant differences in startup times and peak power outputs. Lactosides were found to inhibit the development of biofilms when compared to bare graphite. Mannosides were found, instead, to promote exoelectrogenic biofilm adhesion and anode colonization, a finding that is supported by quartz crystal microbalance experiments in inoculum media. These differences were observed despite both adlayers possessing thickness in the nm range and similar hydrophilic character. This suggests that specific glycan-mediated bioaffinity interactions can be leveraged to direct the development of biotic electrocatalysts in bioelectrochemical systems and microbial fuel cell devices.

## 1. Introduction

The accumulation of greenhouse gases in the atmosphere coupled to the world’s energy consumption rate is fueling a scientific and economic drive to increase exploitation of alternative renewable and sustainable energy sources. Oil will not appreciably run out for at least 100 years or more; however, worldwide energy consumption is projected to double by the year 2050 and triple by 2100, and this could increase pressure on oil reserves and anticipate their depletion up to 20 years earlier than currently expected [1,2,3]. In addition to this, population growth is contributing to carbon emissions, waste generation, and their associated energy demands. For instance, it is estimated that agricultural activities alone are responsible for 14% of the total global anthropogenic greenhouse gas emissions [4], while wastewater processing via traditional treatments requires 0.3–0.6 kWh/m^3^ of treated effluent [5].

**Figure 1:**
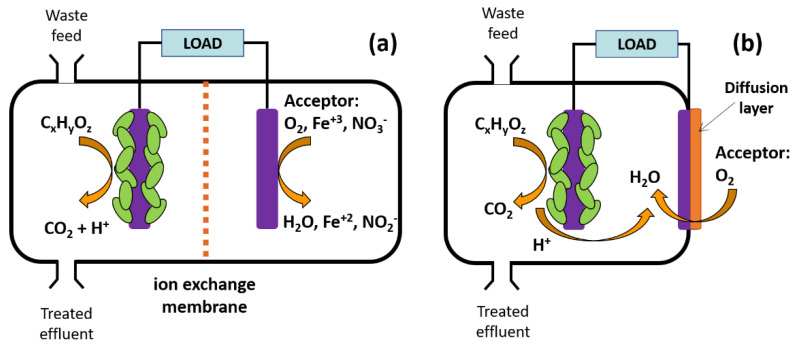
Schematic of a microbial fuel cell in the (**a**) double-chamber and (**b**) single-chamber configurations for the oxidative treatment of organic compounds from waste coupled to power generation through an external circuit. Electrocatalysis at the anode takes place thanks to the formation of an electroactive biofilm that can transfer electrons to the electrode serving as a current collector.

Microbial fuel cell (MFC) technologies could contribute new solutions, particularly in the context of the NextGenerationEU program [6], as they enable sustainable utilization of resources and improved valorization of waste streams [7,8,9]. MFC devices exploit the ability of specific bacteria called exoelectrogens to oxidatively metabolize organic matter and transfer electrons during the respiration process outside their cellular membrane to a solid electrode through a cascade of redox reactions [1,2,10]. Figure 1 shows typical examples of MFC device implementation using two-chamber and single-chamber setups for the processing of organic compounds coupled to power generation. MFCs are attractive because they can potentially combine waste treatment with the production of electricity, thus offering energy-efficient wastewater processing as well as new routes for the valorization of biowastes via energy harvesting [11]. However, their applications are still hampered by the complexity of device scale up, low power outputs, poor stability, and the need to limit component costs to improve their techno-economic profile [11,12,13,14,15,16].

The development of anodic exoelectrogen biofilms with effective electronic coupling to the solid electrode support is critical to high-efficiency MFC performance, as low-resistance coupling is necessary to achieve fast charge transfer and oxidative bioelectrocatalysis [16,17]. Carbon-based materials are widely used as bioanode supports because of their high conductivity, good mechanical and chemical stability, and low cost, and there has been great interest in controlling their properties to improve the biofilm-carbon interface for MFC applications. Carbons/nanocarbons can be fabricated with a range of different properties that make them attractive for electrochemical applications [18], and it has been previously demonstrated that MFC performance can be enhanced through modifications of both their morphology/nanostructure and their surface chemistry [15,16,19,20]. The influence of anode roughness on biofilm colonization has been reported to be crucial for electrocatalytic microorganisms; biofilm attachment is facilitated by electrode cavities while ohmic losses can be minimized by increasing surface area [20]. Power density performances can be enhanced if carbon morphology is modified to achieve high specific surface area [20] and this strategy has been shown to result in fast colonization times in aerobic reactors, as reported by several groups [21,22,23,24]. High wettability has also been observed to result in faster biofilm development and different electrode modification treatments have been explored to this end. Oxidative treatments that increase hydrophilicity of the carbon surface [15,25,26,27] and thermal treatments of graphite and carbon felts in ammonia atmosphere [15,28,29] are among those shown to enhance adhesion and growth of biofilms and final device performances.

Several reports, including by our group, have also used functional thin films to better understand the role that surface charge and specific functional groups play in regulating biofilm colonization at bioanode surfaces [30,31,32,33,34,35,36]. Santoro et al. [36] and Şen-Doğan et al. [35] have investigated the role of surface functionalities using self-assembled monolayers of alkyl/arylthiols on gold anodes and they have found that functional layers with ionizable groups can affect startup times and regulate the composition of the bioanode film. On carbon-based materials, aryldiazonium cation chemistry has been used by several groups to deposit well-defined molecular adlayers aimed at investigating the role of charged acid/base groups [30,31,32,34] and hydrophilic moieties [32]. These studies have shown that positively charged groups as well as increased hydrophilicity result in improved bioanode colonization and performance at carbon electrodes.

Interestingly, surface functionalization can also be used in MFC applications to exploit specific bioaffinity interactions for controlling bioelectrocatalysis. Lapinsonnière et al. [33] elegantly demonstrated that grafting of phenylboronic pinacol ester diazonium cations onto carbon electrodes accelerates bioanode colonization and MFC startup. This result was attributed to the ability of boronic acids to bind lipopolysaccharides in bacterial membranes and consequently enhance the affinity between bacteria in the inoculum and the solid current collector; it was noted nonetheless by the authors that both electroactive and electroinactive bacteria might be recruited to the surface via this route. Work from our group using aryldiazonium grafting of phenyl-mannoside adlayers onto carbon anodes also showed that surface functionalization with glycans that are specifically recognized by lectins in bacterial pili and fimbriae [37,38,39] results in accelerated MFC startup times and peak power outputs [14]. This result suggests that electrode functionalization with selected glycans could be used to signal cell adhesion and might offer a route to selectivity during anode colonization.

It is well known that glycans can play a dual role in biological process of both promoting and inhibiting cell adhesion, in the first case by enabling specific lectin recognition processes while in the second by weakening unspecific interactions, as occurs in the cell glycocalyx [40]. Work from our group has provided strong evidence that glycan functionalization via aryldiazonium chemistry can be leveraged to reduce unspecific binding in biomass-rich media in the case of glycans that are not specifically recognized. In particular, we have shown that the immobilization of phenyl-lactoside adlayers onto a range of carbon, metals, and polymeric substrates can be used to reduce protein adsorption and impart biofouling resistance in both the laboratory and field settings [41,42,43,44]. The benign environmental and toxicity profile of carbohydrates, coupled with their excellent chemical stability towards oxidation and their low cost, make them ideal for applications requiring long-term fouling resistance in the absence of biocidal effects. Nonetheless, applications of glycan modifications to fouling control in MFC devices remain unexplored.

Although biofilm development is desirable for bioelectrocatalysis at MFC anodes, adventitious colonization of other components is instead highly detrimental. For instance, biofilm formation at membranes in two-chamber devices [45,46] or at the abiotic cathode in single-chamber MFCs [40,47,48] is undesirable and can compromise performance. Likewise biofilm overgrowth and unwanted fouling can also be responsible for anode degradation leading to a decrease in electrode coupling and a reduction of the electroactive anode area [11]. Therefore, it would be ideal to develop new electrode modification strategies that can promote electroactive biofilm development at the anode side while inhibiting adventitious fouling in the biomass-rich MFC electrolyte. Such strategies could offer possible routes to achieving long-term performance stability, particularly in challenging waste treatment applications of MFCs, and would improve the ability to selectively recruit desirable bacterial communities. Importantly, any fouling inhibition strategies in MFC applications cannot rely on the use of biocides or toxic leachates, as these can compromise the all-important formation of the electroactive biofilm. This suggests that glycan functionalization of electrodes and components might be a promising and versatile route that can be used to select, promote, and/or inhibit biofilm formation in MFC applications.

**Scheme 1:**
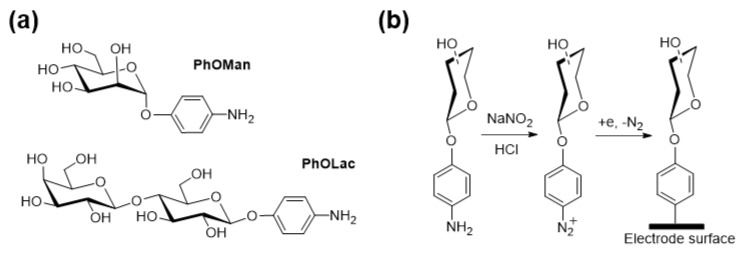
(**a**) Aminophenyl precursors bearing lactoside and mannoside moieties used in our experiment: 4-aminophenol-α-d-mannopyranose (PhOMan) and 4-aminophenol-*O*-β-d-galactopyranosyl(1→4)-β-d-glucopyranoside (PhOLac). (**b**) Diazotization reaction followed by electrochemical grafting of aryldiazonium cations onto electrode surfaces.

In this work, we evaluate for the first time the pro-fouling effect of functional thin films prepared from aryldiazonium using 4-aminophenol-α-d-mannopyranose and 4-aminophenol-*O*-β-d-galactopyranosyl(1→4)-β-d-glucopyranoside precursors (Scheme 1) and grafted at anodes of MFCs inoculated with common anaerobic wastewater. Lactosides and mannosides are known to regulate specific and non-specific interactions and their uptake through the cell membrane has been widely studied in the past [49,50,51]. Covalent attachment onto carbon electrodes was achieved via electrochemically assisted grafting. We first discuss the effect of the different functionalization protocols on electrode roughness and wettability. The electrochemical response of modified surfaces was tested in MFC devices prepared using anaerobic wastewater and phosphate buffered saline as anodic inoculum. We evaluated the influence of phenyl-glycoside adlayers on the anodic MFC graphite rod electrodes on long-term operation time (more than two months) and we discuss these results on the basis of the power output and overall electrochemical MFC performances. Studies of surface selectivity towards the wastewater inoculum using quartz crystal microbalance demonstrate that the choice of glycan has a profound effect on interfacial interactions with bacteria in the biomass-rich MFC electrolyte.

## 2. Results

### 2.1. Surface Functionalization Using Phenyl-Glycosides

Organic adlayers of phenyl-lactosides and phenyl-mannosides were immobilized on conductive substrates using electrochemically assisted grafting from aqueous solutions. The aryldiazonium salts of 4-aminophenol-α-d-mannopyranose (PhOMan) and 4-aminophenol-*O*-β-d-galactopyranosyl(1→4)-β-d-glucopyranoside (PhOLac) were prepared via diazotization with NaNO_2_ in HCl solutions (Scheme 1), as previously reported [42,44]. X-ray photoelectron spectroscopy and infrared reflectance spectroscopy have previously been used to characterize the molecular structure of glycoside films prepared from the same precursors via spontaneous grafting and 300 s potential steps, and in both cases results show that the grafting process leads to immobilization of intact phenyl-glycoside moieties [41,42,43,52]. In this work we therefore focus specifically on the characterization of properties relevant to the electrode preparation protocol for electrodes used in MFC device tests.

Two different conductive substrates were used to characterize the adlayers prepared via 35 s potential steps: indium tin oxide (ITO) and graphite rods (GR). ITOs were used as supports for AFM roughness and layer thickness determinations whereas GRs were used for all MFC experiments and for contact angle characterizations, as discussed below. A 35 s potential step was used to reductively graft phenyl-glycosides; the total integrated charge density obtained via normalization by the exposed geometric area and associated with the generation of aryl radicals during the potential step experiments for all samples is reported in Table 1. Results show that for both ITO and GR, the total charge is well in excess over the value required for monolayer grafting (10^−6^–10^−5^ mol m^−2^ corresponding to 0.1–1.0 C m^−2^) [52,53]. Integrated charges on GR substrates were more than an order of magnitude larger than those obtained with ITO surfaces; this is likely due to the greater microroughness displayed by polished GR surfaces (rms 61 ± 16 nm) [14] compared to the ITO substrates (see below).

**Figure 2:**
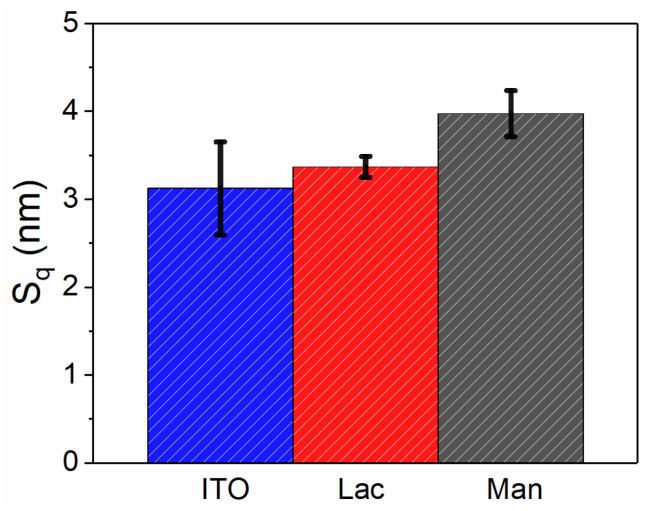
Determinations of rms roughness values (S_q_) for bare ITO (blue), ITO_Lac (red), and ITO_Man (grey) samples.

Figure 2 shows the effect of adlayer modification on electrode surface roughness. The rms roughness values after surface functionalization are similar to those of the bare substrate, thus indicating that the modification protocol leads to conformal adlayers. A slight decrease in the roughness error bars can however be observed for modified ITO surfaces, suggesting that adlayers might smooth out substrate asperities as previously observed for glycan thin films prepared via spontaneous reaction of the aryldiazonium cations [44].

**Figure 3:**
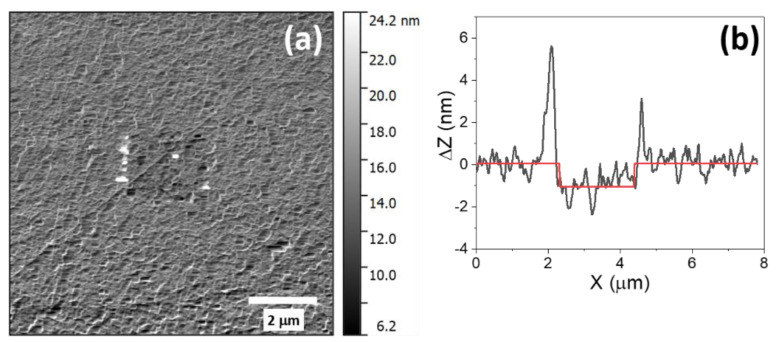
(**a**) ΔZ difference image obtained via AFM of an ITO_Lac surface before and after removal of a 2 × 2 μm^2^ region of the organic film. (**b**) Average height profile extracted from the ΔZ image in (a).

The thickness of the layers was investigated using difference topography images obtained via AFM [42,44,54,55]. Figure 3a shows a height difference image (ΔZ) of an ITO_Lac layer grafted with a 35 s potential step. The image clearly shows a rectangular region of height depression corresponding to the 2 × 2 μm^2^ raster pattern created by the tip in contact mode, as described in detail in the experimental section. A control experiment carried out using the same deflection setpoint on a bare surface (see Supplementary Materials) shows that identical conditions do not result in removal of ITO. Therefore, the rectangular depressed region observed in the ΔZ images can be confirmed to be the result of removal of the organic functional film. Figure 3b shows the average height profile extracted from the ΔZ image. The estimated thickness of the glycan layer of ITO_Lac was found to be 1.1 ± 0.8 nm; this mean height is larger than the value of 0.8 nm obtained for spontaneously grafted monolayers of phenyl-lactoside, but lower than 1.9 nm obtained samples using a 300 s potential step [42]. Similar measurements for ITO_Man layers are shown in the Supplementary Materials. In the case of ITO_Man, the height step is at the limit of detection; nonetheless, a step of ca. 0.5 nm can be qualitatively discerned. This estimate is in good agreement with layer thicknesses obtained after spontaneous grafting with monosaccharides [44], which suggests that 35 s potential step yields a sparse conformal mannoside adlayer.

**Figure 4:**
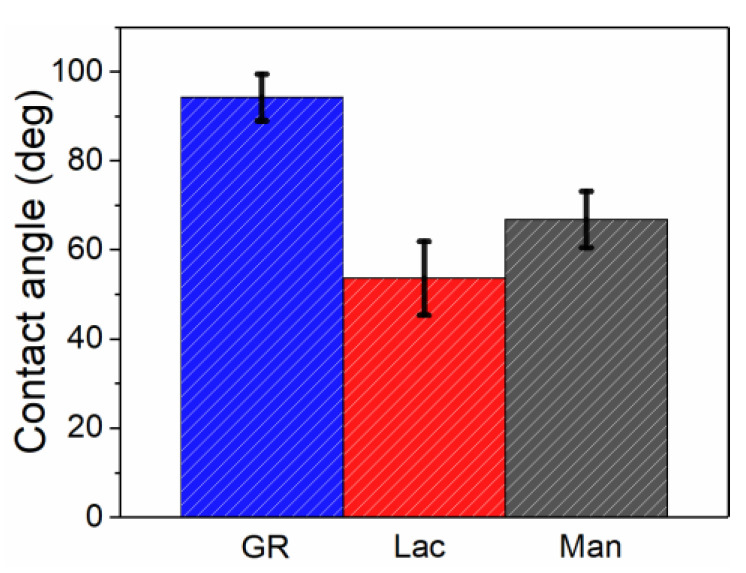
Water contact angle determinations of bare GR (blue), GR_Lac (red), and GR_Man (grey) electrodes used for MFC device testing.

MFC devices can use a range of carbon materials as current collectors at the anode: graphite rods (GR), graphite fiber brushes, carbon cloth, carbon paper, carbon felt, and reticulated vitreous carbon have all been investigated [10,56]. In our case, we chose GR as the electrode material for both anode and cathode compartments due to its low cost, low resistivity, and versatility [56], and because results obtained on these materials can be easily generalized to other low-cost carbons. Figure 4 shows water contact angle determinations at bare GR and GR_Lac and GR_Man samples. Results show that independently of the type of glycoside, the electrochemical functionalization via potential step leads to an increase in surface wettability: the water contact angle was found to decrease from 94° ± 5° for bare GR [14] to statistically similar values of 67° ± 6° and 54° ± 8° for GR_Man and GR_Lac, respectively.

**Figure 5:**
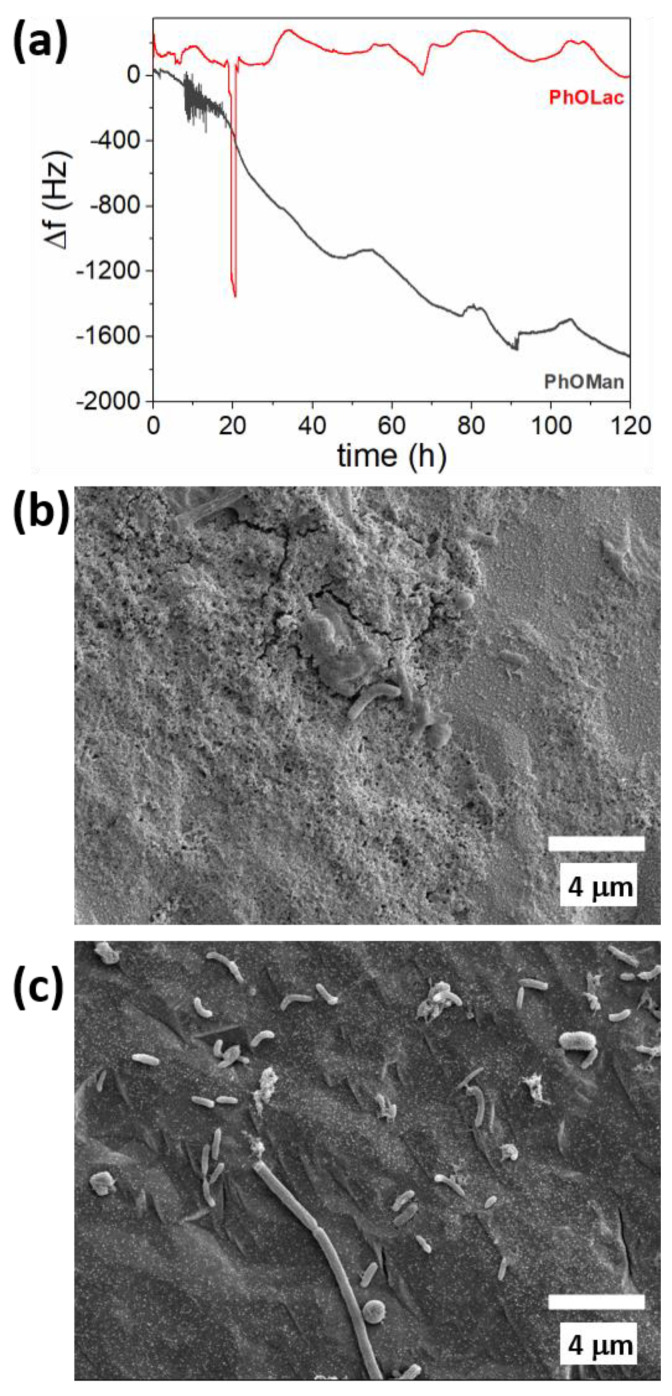
(**a**) Change in resonant frequency of QCM carbon-coated crystals functionalized with PhOLac and PhOMan adlayers as a function of time immersed in a dilute wastewater inoculum; frequency transients observed over the first day (e.g., at 20 h) are attributed to residual sedimentation in the wastewater inoculum. SEM images of the biofilms formed on PhOMan (**b**) and PhOLac (**c**) at the end of the 5 day immersion are also shown.

Wettability is a property that is critical both for the ability of a surface to resist biofouling [44,57,58] and for the promotion of biofilm development in MFC [32,59]. The development of GR surfaces with similar wettability but different glycoside termination such as GR_Lac and GR_Man is of particular interest, as it enables one to discriminate the role of specific surface-cell bioaffinity interactions arising from glycoside-lectin binding, without potential confounding effects arising from changes in hydrophilicity. A first insight into the role of specific glycosides in promoting biofilm development during MFC startup was obtained from QCM experiments shown in Figure 5 [60]. Carbon-coated QCM crystals were functionalized with phenyl-lactoside and phenyl-mannoside monolayers via spontaneous grafting (2 h immersion in 1 mM solution) [61]. The crystals were thereafter immersed in a decanted inoculum solution used for MFC studies, which contained 50% *v*/*v* of wastewater in Na_2_HPO_4_/NaH_2_PO_4_ buffer solution, as described in the experimental section. Sodium acetate was added as a nutrient to a 0.012 M concentration and the frequency of the crystal was thereafter monitored over 5 consecutive days to investigate the rate of biofilm development under conditions similar to those experienced in the early stages of MFC startup. Figure 5a shows changes in resonant frequency observed for PhOLac and PhOMan functionalized carbon crystals immersed in the inoculum. The data clearly show that greater changes in frequency are observed in the case of PhOMan functionalized carbon; for these surfaces, the frequency shows a progressive decrease over the 5 days, whereas for PhOLac surfaces, the frequency shows slow oscillations of ca. ±100 Hz, consistent with temperature fluctuations of ca. 10 ppm over the 24 h cycle [62]. The result of Δ*f* measurements carried out over 5 days for replicate samples and inoculum solutions are summarized in Supplementary Materials. The mean Δ*f* changes observed for bare carbon surfaces are intermediate relative to those on PhOMan and PhOLac functional layers.

The total change in resonant frequency Δ*f* for a crystal immersed in a liquid medium can result from multiple contributions so that $\Delta f=\Delta f_{m}+\Delta f_{y}+\Delta f_{a}+\Delta f_{x}$ [63,64,65], where $\Delta f_{m}$ is the frequency shift resulting from a mass increase at the crystal surface, $\Delta f_{y}$ is the shift arising from viscous damping, $\Delta f_{a}$ results from surface stress, and $\Delta f_{x}$ is due to non-shear coupling. $\Delta f_{a}$ and $\Delta f_{x}$ are typically time-independent and therefore should not be responsible for the change observed over the 5 day incubations. Therefore, the changes can be attributed to a combination of mass accumulation at the surface and increased viscous damping associated with the adsorbed mass, which is consistent with progressive cell adhesion and biofilm formation on the surface of quartz resonators [66,67,68]. Results therefore suggest that biofilm formation occurs rapidly on surfaces modified with PhOMan, whereas for PhOLac functionalization, the surface colonization is inhibited or delayed under similar conditions.

This conclusion is further supported by SEM imaging of the QCM crystals obtained after the 5 day immersion tests, shown in Figure 5b,c. The images show significant differences in biofilm quality between the two electrodes: PhOMan-coated electrodes develop a smooth and thin continuous biofilm (Figure 5b) whereas PhOLac surfaces show inhomogeneous bacterial adhesion and bare carbon regions. Crystals with bare carbon surfaces yielded intermediate behavior between those of PhOMan and PhOLac, as shown in the Supplementary Materials, in agreement with Δ*f* determinations. This result suggests that functionalization with similarly hydrophilic adlayers can lead to drastically different biofilm development on carbon surfaces and that the identity of the glycoside used during functionalization is likely to play a major role in determining colonization rates and/or biofilm morphology. To test whether these effects at early colonization stages also translate into differences in MFC performance, we carried out long-term experiments using MFC devices, as described in the following section.

### 2.2. MFC Device Testing with Glycan-Modified Bioanodes

**Figure 6:**
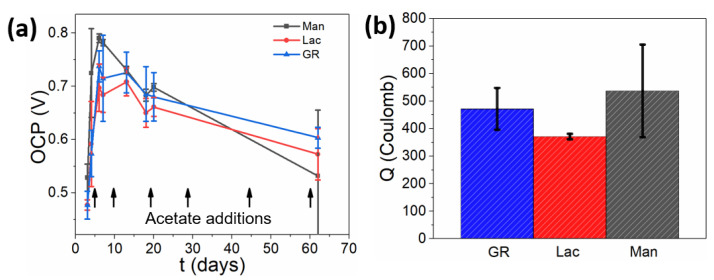
(**a**) Open-circuit potential (OCP) average of all MFC devices tested as a function of time. Black arrows indicate when fresh solution (PBS + sodium acetate) was added to the anode compartment as nutrient. (**b**) Total integrated charge obtained from the MFC devices over the duration of the experiment (1000 Ω load).

The influence of electrografted glycosides on electroactive biofilm formation was evaluated by testing the different coated graphite rods as bioanodes in double-chamber MFC devices [14]. All cells possessed identical geometry and the measurements were performed in triplicate configuration: three cells equipped with each of GR_Lac and GR_Man, as well as three additional ones with bare polished GR (GR_bare) as control were set up and started up at the same time. All 9 MFC devices were inoculated with 50 vol.% wastewater in PBS with 0.012 M sodium acetate [69], and their performance was monitored over the course of 2 months. Figure 6a shows the open-circuit potential (OCP) of the cell as a function of time. At regular intervals indicated by the arrows in the figure, the anolytes were replenished for nutrients, by replacing half of their volume with 0.012 M sodium acetate in PBS. The OCP oscillated between 0.7 and 0.8 V after 6 days, which is comparable with values reported from the literature for cells fed with sodium acetate (oxidized by *Geobacter*–dominated biofilms at ca. −0.5 V vs. AgCl/Ag) at the anode and ferricyanide as electron acceptor at the cathode (reduced at ca. +0.2 V vs. AgCl/Ag) [10,70,71,72]. Up to day 20, the OCP follow the order GR_Man > GR_bare > GR_Lac. However, the open-circuit voltage gives an overview of the thermodynamic potential developed by the bioanode, it does not take into account any internal current-dependent overpotential losses in the MFC: (i) activation losses; (ii) bacterial metabolic losses; and (iii) mass transport or concentration losses [1,10]. A better indication of overall MFC performance is provided by the total charge passed under load conditions (1 kΩ), which is shown in Figure 6b. Voltage @1 kΩ vs. time trends for all cells used to calculate integrated charges is reported in the Supplementary Materials. The data suggest that GR_Lac anodes inhibit development of the microbial community, yielding the lowest acetate conversion rate. Importantly, one of the GR_Lac-equipped MFC devices (see Supplementary Materials) did not show development of an output voltage and was excluded from the average reported in Figure 6b. Although the precision was too low to establish statistical difference, the trends in mean charge values suggest that phenyl-mannoside adlayers lead to faster biofilm development, whereas phenyl-lactoside adlayers have an inhibitory effect and bare graphite appears to yield intermediate performance. Therefore, complementary characterization using power density curves and voltammetry was carried out to support this initial observation.

**Figure 7:**
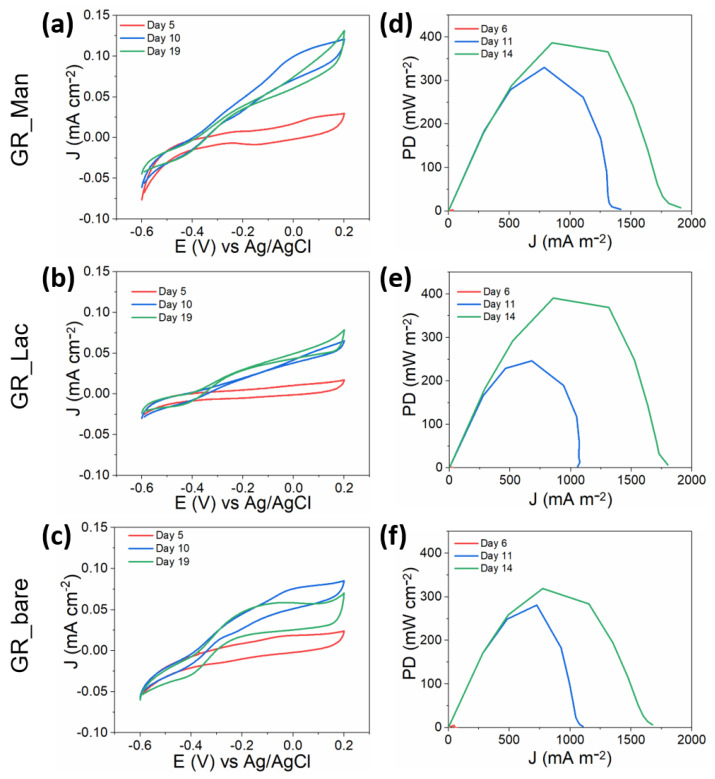
In situ CVs of graphite anodes of MFC devices equipped with GR_Man (**a**), GR_Lac (**b**), and GR_bare (**c**) anodes. CVs were obtained at 5 mV s^−1^ after day 5, day 10, and day 19 from the inoculum injection. The corresponding power density curves obtained at day 6, day 11, and day 14 for MFC devices equipped with GR_Man (**d**), GR_Lac (**e**), and GR_bare (**f**) are shown on the right hand side.

To better understand bioanode development, bioelectrocatalytic activity was investigated via cyclic voltammetry. Figure 7a–c show representative cyclic voltammograms of GR_Man, GR_Lac, and GR_bare anodes at 5, 10, and 19 days after startup. After 10 days, all of the anodes show a catalytic waveform associated with acetate oxidation at ca. -0.4 V, thus indicating a mature biofilm development for all of the considered samples [31]. The current density for GR_Man anodes is greater than for GR_Lac at either day 10 or 19; this is also confirmed for the other replicates (Supplementary Materials). The power density curves shown in Figure 7d–f are in close agreement with cyclic voltammetry data. After 6 days, the maximum power density obtained for all cells is extremely low and observed at negligible current densities (Table S1), while after 11 days all cells show power density profiles that indicate that bioanode development has taken place. At day 11, the MFC with GR_Lac1 reports a peak power density of ca. 270 mW m^−2^ which is lower than GR_Man1, which already shows >300 mW m^−2^ peak power. Replicates of cyclic voltammetry and power density curves shown in Supplementary Materials indicate that GR_Lac samples yield poor exoelectrogenic biofilm development. We note that for one of the cells, it was not possible to obtain a power density profile after 19 days of operation, as this cell developed a mature biofilm only after 30 days, showing an anodic catalytic waveform only at day 31 (see Supplementary Materials).

In summary, data show that the affinity of the biofilm towards the anodic surface is affected by the presence of different types of glycosides, with mannosides resulting in enhanced startup times and bioanode development, and lactosides leading to delayed bioanode maturation.

## 3. Discussion

In this study we tested the effect of aryldiazonium functionalization with phenyl-lactoside and phenyl-mannoside groups when used to coat carbon electrodes in order to obtain a better understanding of how these saccharides influence biofilm adhesion processes, and to investigate their application as biofilm promoters/inhibitors in MFC devices. Modification with both types of aryl layers offers an effective route to change the wettability of the anode materials, and changes in contact angle resulting from modification were similar for both types of glycosides. However the specific glycoside structure appears to play an important role in MFC performance. Interestingly, the accelerated startup observed for GR_Man samples is likely specific to the mannoside structure and not attributable simply to an increase in the hydrophilic character of the graphite surface after functionalization. In fact, functionalization with lactosides, yielding a similarly hydrophilic surface, was found to result in an inhibitory effect towards biofilm development in MFC devices. These results are in agreement with trends in surface adsorption in the presence of wastewater inoculum observed via QCM measurements, which showed that adsorption is inhibited on phenyl-lactoside-modified surfaces when compared to phenyl-mannosides. This difference in adsorption at early stages of biofilm formation readily translates into improved bioelectrocatalysis performance of acetate at GR_Man at day 10 relative to GR_Lac and GR_bare. Similarly, it is reflected in the trends in peak power density of MFC devices at day 11, which show ~25% higher outputs for MFC devices equipped with GR_Man than with GR_Lac anodes.

Inhibition of bacterial growth by lactose has been clearly documented in the literature, but the specific effect on MFC biofilm communities is unprecedented in the literature. Lactose influence on *Klebsiella oxytocu B 199* growth rates shows slow growth as reported by Straight et al. [73]. In this work, they examined the growth rates in the presence of mono- and di-saccharides (glucose galactose and lactose) and the effect of their concentration in the *Klebsiella oxytocu* inoculum. It has been demonstrated that, in the presence of a mixed glucose/lactose medium, the lactose metabolism was dependent on the glucose concentration and lactose transport started only when the growth on glucose was completed. On the contrary, in the case of a mixed galactose/lactose medium, the consumption of galactose was performed during the first exponential growth phase and the lactose was consumed in a second growth phase. Strains of *E. coli*, which is known to exhibit electrogenic activity, ref. [1] appear also to be sensitive to lactose addition, as reported by Hofsten [74]. Following the intracellular galactose regulation theory, the authors report the inhibition of culture grown on *E. coli* strains that possess an effective transport system for β-galactosides and contain high amounts of β-galactosidase after the lactose addition. This finding was ascribed to cell permeability properties for nutrient uptake and/or to an inhibition process caused by the lactose excess on β-galactoside permease enzyme. Similar results have been reported by Inada et al. [49]. In their work, *E. coli* β-galactosidase expression was inhibited when a combination of glucose/lactose was present. In agreement with these observations, our MFC and QCM experiments show that the presence of a phenyl-lactoside adlayer inhibits biofilm development.

Mannose is known to be recognized by FimH adhesin of *E. coli.* [37,75]. The effect of the mannose and glucose consumption has been evaluated previously by Lawford and Rousseau [51], who have shown that in the presence of mannose and glucose (2:1 mass ratio), the mannose consumption by *E. coli* is not affected by the glucose but on the contrary it produces a slightly enhanced rate of mannose utilization. A mixture of glucose and one of the so-called “A-sugars” (glucose, fructose, mannitol, mannose, and sucrose) seems to not provide a diauxic lag in *E. coli* and *B. subtilis* but, on the contrary, they are consumed simultaneously [76]. In our work the thin layer of produced mannose appears to signal the adhesion of an exoelectrogenic biofilm community, as shown by the biocatalytic waves in Figure 7.

As regards the effect of electrode modifications on MFC performance, three main factors should be considered. First, the modification should produce an electrode interface that is not only pro-biofouling to favor quick and thick catalytic biofilm development but also at the same time the modifier layer should be designed to maintain an efficient electrical connection between the electroactive biofilm and the electrode. The former point can be explored by different strategies such as tuning some of the following factors at the interface: wettability, electrostatic and/or bioaffinity properties, while the latter can be addressed by controlling the thickness of the modifier and/or its intrinsic electronic conductivity. In addition, to improve the performance of any fuel cell, identification of the critical limiting factor is of utmost importance (catalysis at the anode or cathode, internal resistance, mass transport, etc.). In our study, the cathodic reaction was not limiting due to the presence of a quasi-constant high amount of oxidant (ferricyanide) in the catholyte.

In light of the differences observed, we propose that wettability is less important a factor than the structure of the saccharide involved in the functionalization, which instead contributes to the regulation of bioanode development by eliciting different enzymatic responses from the exoelectrogenic microorganisms. Further studies ex situ on the type and role of the bacteria community that colonizes the electrode surface would be important to better evaluate the role of these saccharides on MFC performances.

## 4. Materials and Methods

### 4.1. Materials 

Sodium phosphate monobasic (ReagentPlus ≥ 99%), sodium phosphate dibasic (BioReagent ≥ 99%), sodium acetate (BioXtra ≥ 99%), potassium chloride (BioXtra ≥ 99%), and ammonium chloride (≥99.5%) were all purchased from Sigma Aldrich (Arklow, Ireland). Potassium hexacyanoferrate(III) (ACS reagent ≥ 99%) was purchased from Fluka (Dublin, Ireland). Glassy carbon (GC) disk electrodes were purchased from HTW (Sigradur, Ø0.50 cm; Thierhaupten, Germany)); indium tin oxide (ITO) coated glass (7 Ω per square) was purchased from Xinyan Technology Ltd. (Kowloon, Hong Kong); graphite rods (GR) were purchased from Morgan Carbon (Eguisheim, France). PET-reinforced Fumasep FTAM-E anion exchange membrane was purchased from Fumatech BWT (Bietigheim-Bissingen, Germany).

### 4.2. Surface Functionalization 

Electrode modifications were carried out in 1.0 mM aqueous solutions of the aryldiazonium cations prepared from 4-aminophenol-α-d-mannopyranose (PhOMan) and 4-aminophenol-*O*-β-d-galactopyranosyl(1→4)-β-d-glucopyranoside (PhOLac) after diazotization (Scheme 1). The precursors were synthesized, isolated, and diazotization was carried out in situ immediately prior to functionalization, as described in detail in previous work [14,42,52,77]. Substrates investigated were functionalized via electrochemical reduction of the aryldiazonium salts in a 3-electrode cell, using the substrate as the working electrode, a Pt wire as counter electrode, and Ag/AgCl as reference. The geometric working area was defined each time using Teflon tape; a 35 s potential step was applied at −0.60 V and −0.65 V vs. Ag/AgCl for functionalization with PhOLac and PhOMan adlayers, respectively [14]. After modification, both supports were rinsed with water, sonicated for 1 min in methanol, and rinsed with water again prior to further analysis or testing in MFC devices [42].

### 4.3. Characterization 

Electrochemical experiments were carried out using Metrohm Autolab potentiostats (PGSTAT 204 and 302N). Water contact angle (WCA) measurements were performed using the sessile drop method (FTA1000) [42,43,77]. Atomic force microscopy (AFM, Asylum Research) was carried out using Au-coated cantilevers (NT-MDT); height profiles of glycan layers were recorded using previously described methods [42,44]: first, a topography image was obtained in tapping mode, then a square region of the organic adlayer was removed via contact mode (2 V setpoint), and finally the height step was imaged via tapping mode. Image analysis was carried out using open software (Gwyddion); surface topographies before and after removal of the organic adlayer were first aligned in the *xy* plane, then subtracted to obtain an image of ΔZ values that corrects for the underlying topography of the conductive substrate. The average height of the step edge was determined to calculate the adlayer thickness [42,55]. Quartz crystal microbalance (QCM, 922A Seiko) was used to monitor biofilm formation using AT-cut 9.12 ± 0.03 MHz carbon-coated crystals before and after functionalization with glycan adlayers in inoculum solutions. The inoculum was left to sediment for 2 h prior to QCM experiments to avoid frequency fluctuations due to macroscopic sedimentation; the clear supernatant was then extracted with a syringe and used for QCM experiments in which the crystal was mounted in a Teflon holder with only one face of 0.2 cm^2^ exposed to solution. The frequency was measured every 1 s over 120 h (5 days) under ambient conditions. After QCM experiments, electrodes were removed from the Teflon holder and left overnight in 0.1 M phosphate buffer and 2.5% *v*/*v* glutaraldehyde solutions; finally, they were washed with distilled water and immersed in aqueous solutions containing progressively increasing ethanol concentrations, for 15 min in each solution. After this procedure, the samples were critical point dried and analyzed with JEOL JSM 7100F scanning electron microscope. Further details are reported elsewhere [14,31].

### 4.4. Microbial Fuel Cell Experiments 

Double-chamber MFC devices were fabricated from polycarbonate (85 mL of volume) with a circular membrane port of ∅1.8 cm [14]. The anodic solution consisted of 50% *v*/*v* wastewater (Beaurade Wastewater Treatment Plant, Rennes, France) in phosphate buffer saline (PBS) solution with 0.032 M Na_2_HPO_4_, 0.018 M NaH_2_PO_4_, 6.0 mM NH_4_Cl, and 2.0 mM KCl [69]. Sodium acetate (0.012 M) was added to the anolyte as nutrient; the resulting inoculum was added to each MFC anodic chamber. A standardized Fe(CN)_6_^−3/−4^ cathode was used for all tests to enable comparisons of anodic performance [10]; 0.1 M K_3_[Fe(CN)_6_] in 0.032 M Na_2_HPO_4_ and 0.018 M NaH_2_PO_4_ was used as catholyte in all cases [14]. To test MFC response in situ, the cathode was used as the counter electrode while the anode acted as the working electrode; a saturated calomel electrode (SCE, CH instruments) was used as reference and placed in the anode compartment. Cyclic voltammetry was carried out over the range −0.6–0.2 V vs. SCE at 5 mV s^−1^; figures show the second scan unless otherwise noted. After electrochemical characterization, each cell was sealed again and left in a thermostatic bath (25 ± 0.1 °C) connected to a 1000 Ω resistance. Power density curves were obtained by connecting the cells to a resistance decade box (Elenco): the resistance connected to the cells was varied in the interval 10000–10 Ω and the voltage was recorded for each resistance after 30 min equilibration. Current and power density values were calculated from the voltage outputs recorded with the multimeter (Keithley 2700) [2,14,31,33].

## 5. Conclusions

Surface functionalization of graphite anode materials with phenyl-mannoside and phenyl-lactoside adlayers was found to lead to differences in MFC startup, development, and power performance. Lactoside groups were found to inhibit the development of an exoelectrogenic biofilm when compared to bare graphite electrodes. On the other hand, mannoside-modified graphite promotes biofilm adhesion and anode colonization, as indicated by their superior performances compared to that of bare graphite anodes. These differences were observed despite both adlayers possessing nm thickness or less, and similar hydrophilic character. Work from several groups have used functional thin films to investigate the role of ionizable moieties (e.g., amines, carboxylates) or of functionalities that modulate wettability (hydrophilic vs. hydrophobic); however, to the best of our knowledge, bioaffinity interactions such as those leveraged in this manuscript are largely unexplored. Our results therefore suggest that MFC output is likely to be affected by the biochemical interactions between bacteria and glycoside structure, thus demonstrating that exoelectrogen adhesion can be mediated via specific glycoside selection. This opens the door to investigating the potential role of specific saccharides or oligo-saccharides for proactively selecting the bacterial consortium composition that develops at MFC bioanodes.

## Figures and Tables

**Table 1:** Integrated cathodic charge associated with electrografting experiments on the two different substrates used in this work.

Sample	Cathodic Charge Density (C m^−2^)
ITO_Lac	3.8 ± 0.5
ITO_Man	3.0 ± 0.6
GR_Lac	32 ± 6
GR_Man	85 ± 9

## Data Availability

Data will be deposited and become available in the following public repository: [http://www.tara.tcd.ie/] accessed on 6 August 2021.

## Supplementary Materials

The following are available online. Figure S1: additional AFM images; Figure S2: additional QCM results; Figure S3: additional SEM of biofilms; Figure S4: potential @1kΩ vs. time for all MFC devices; Table S1: peak power densities at day 6; Figures S5–S7: cyclic voltammograms and power output curves for all MFC devices tested at selected time intervals.

## Sample Availability

Samples of the compounds are not available from the authors.
