# Supplementary material for: Controlling the Carbon-Bio Interface via Glycan Functional Adlayers for Applications in Microbial Fuel Cell Bioanodes
Source: Molecules. 2021 Aug 6;26(16):4755. doi: 10.3390/molecules26164755 (PMC8400688; doi:10.3390/molecules26164755)
Supplement: Supplementary file 1 [file molecules-26-04755-s001.zip › molecules-1311160-supplementary.pdf]

Supporting Information for

Controlling the carbon-bio interface via glycan functional adlayers for applications in microbial fuel cell bioanodes

Alessandro Iannaci,[a] Adam Myles,[a] Timothé Philippon,[b] Frédéric Barrière,[b] Eoin M. Scanlan*[a] and Paula E. Colavita*[a]*

a- School of Chemistry, CRANN and AMBER Research Centres, Trinity College Dublin, College Green, Dublin 2, Ireland.

b- Univ Rennes, CNRS, Institut des Sciences Chimiques de Rennes - UMR 6226, F-35000 Rennes, France.

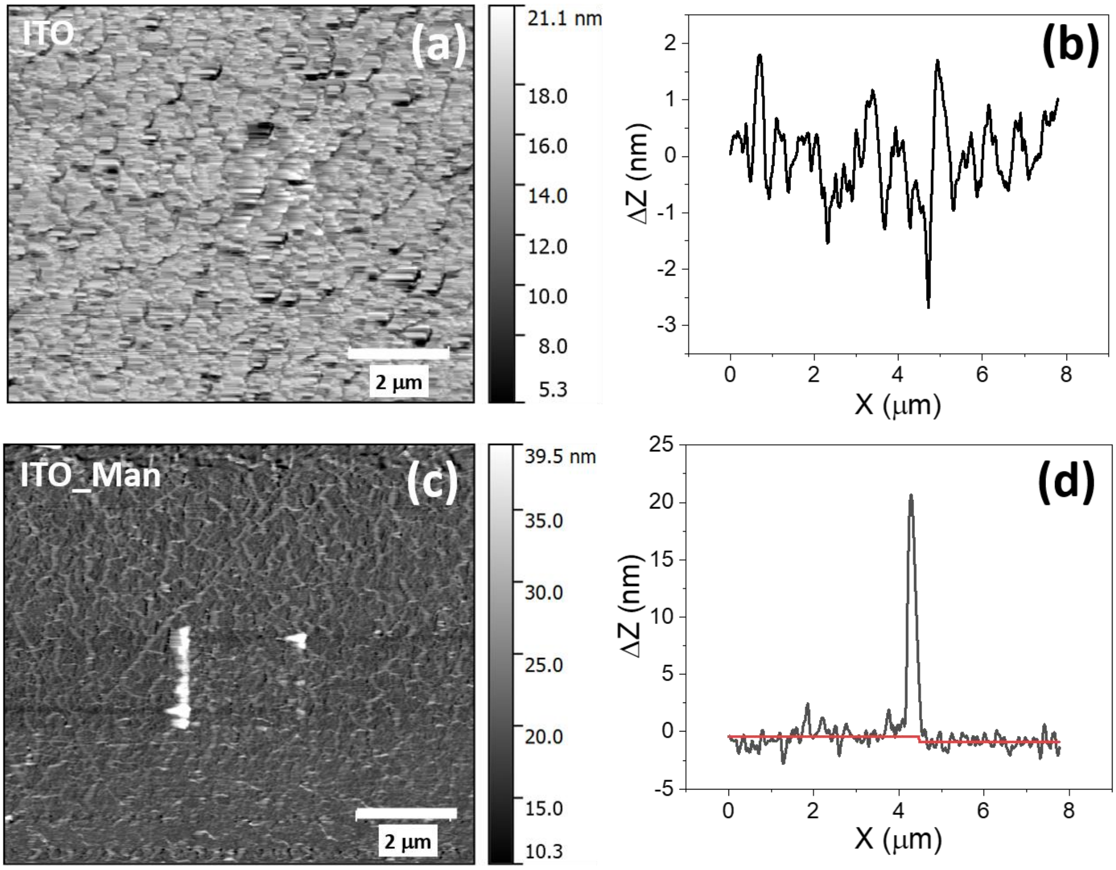

Figure S1. ΔZ difference image obtained via AFM of a bare ITO (a) and of ITO_Man (c) surfaces before and after removal of a 2×2 μm^2 region via raster scanning in contact mode. (a) shows no discernible depressed region, whereas (c) shows a clear region corresponding to removal of the organic adlayer. Average height profiles extracted from the corresponding ΔZ images are shown from bare ITO (b) and ITO_Man (d).

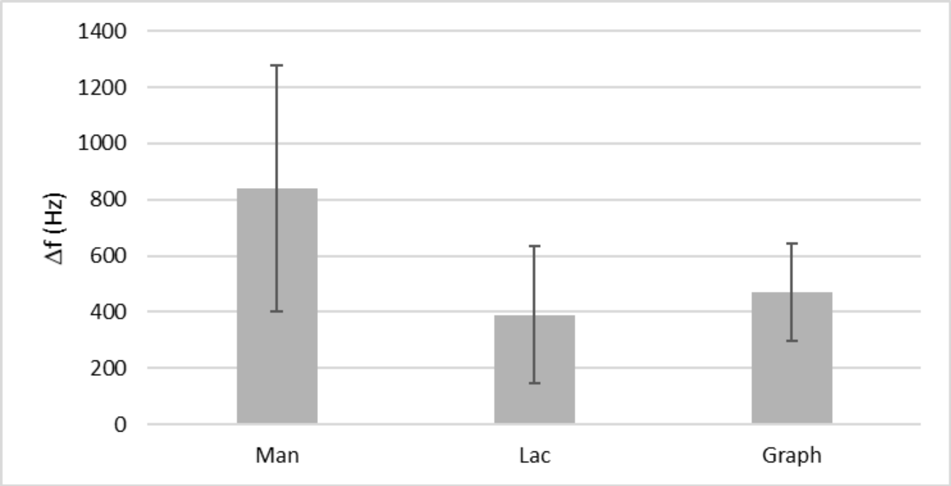

Figure S2. Resonant frequency change measured at QCM carbon-coated crystals bare and functionalised with PhOLac and PhOMan adlayers after immersion in a dilute wastewater inoculum after 5 days, as described in the experimental section; error bars indicate standard errors on the mean calculate from triplicate experiments. Large variances observed are attributed to a combination of temperature effects, differences in the sedimentation of the wastewater inoculum in consecutive experiments and surface condition of pre-coated crystals.

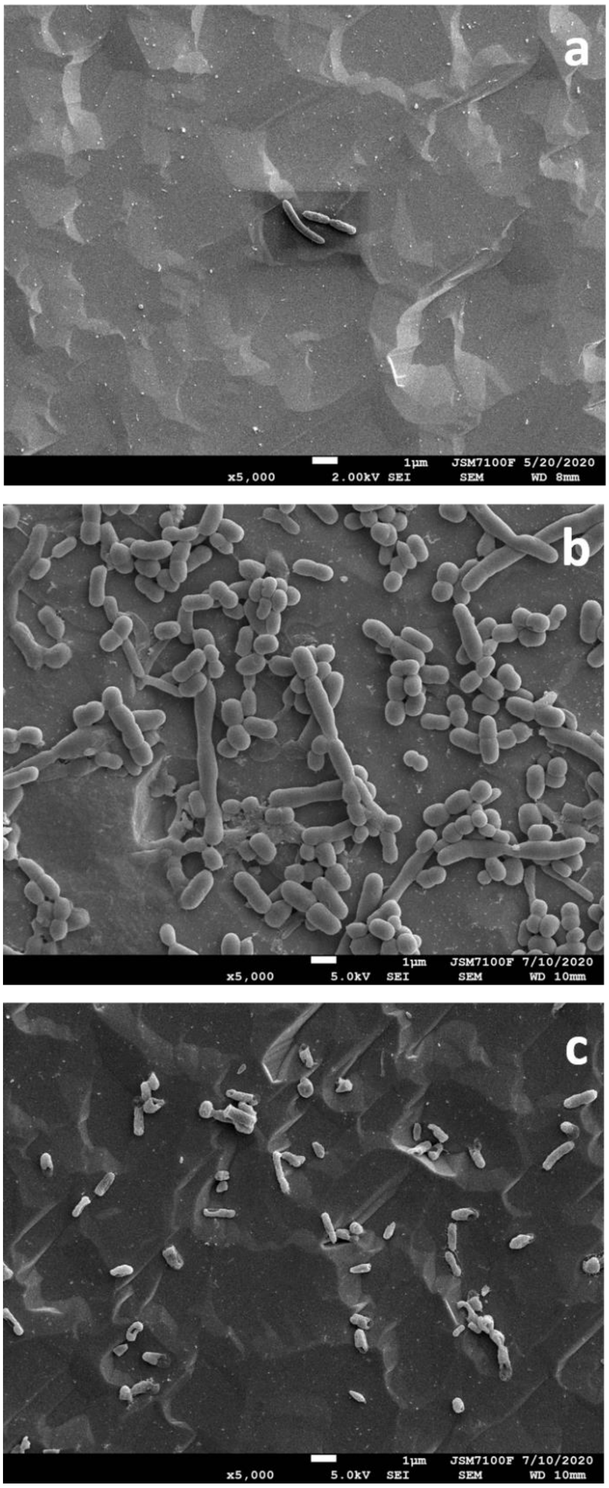

Figure S3. Additional SEM images of carbon-coated crystals functionalised with (a) PhOLac and (b) PhOMan adlayers after both surfaces had been immersed in a dilute wastewater inoculum after 5 days, as described in the experimental section. The image obtained using a bare carbon control at 5 days under identical conditions is shown in (c).

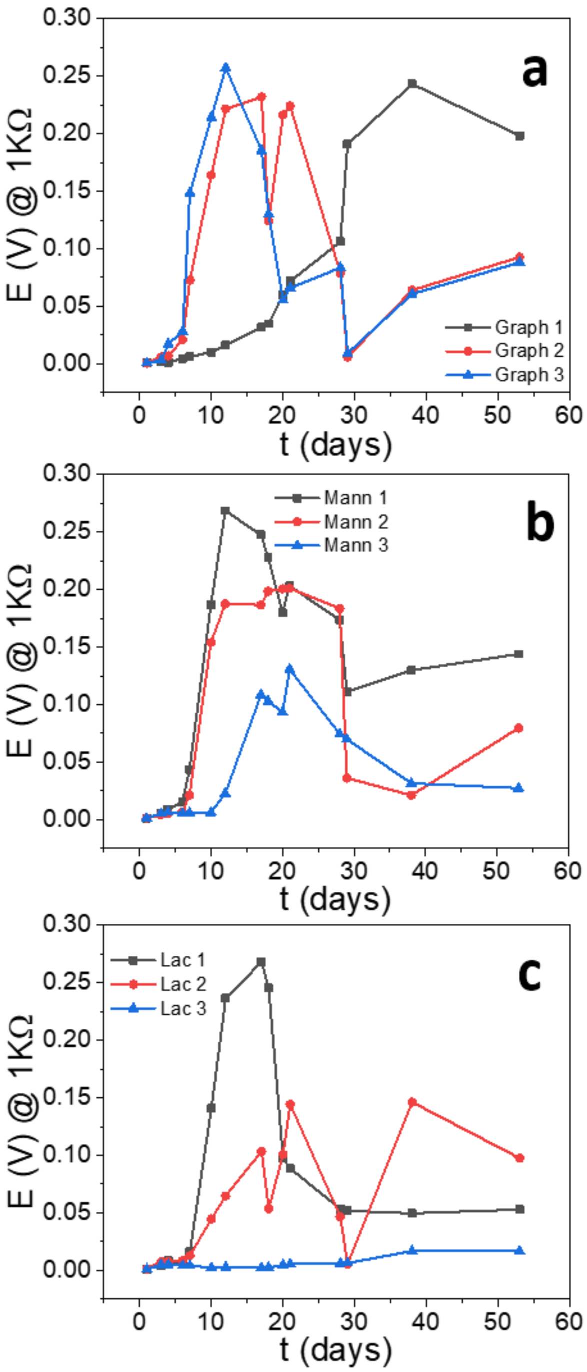

Figure S4. Voltage output obtained when cells were connected at 1KΩ as function of time for all tested MFC devices during the entire period of operation.

Table S1. Maximum power density values recorded at day 6 for MFC devices shown in Figure 7 in the main text.

Sample	Max. power density ($mW\ m^{-2}$)	Current density at maximum ($mA\ m^{-2}$)
GR_Man1	2.75	36.0
GR_Lac1	1.21	23.9
GR_bare1	4.80	47.5

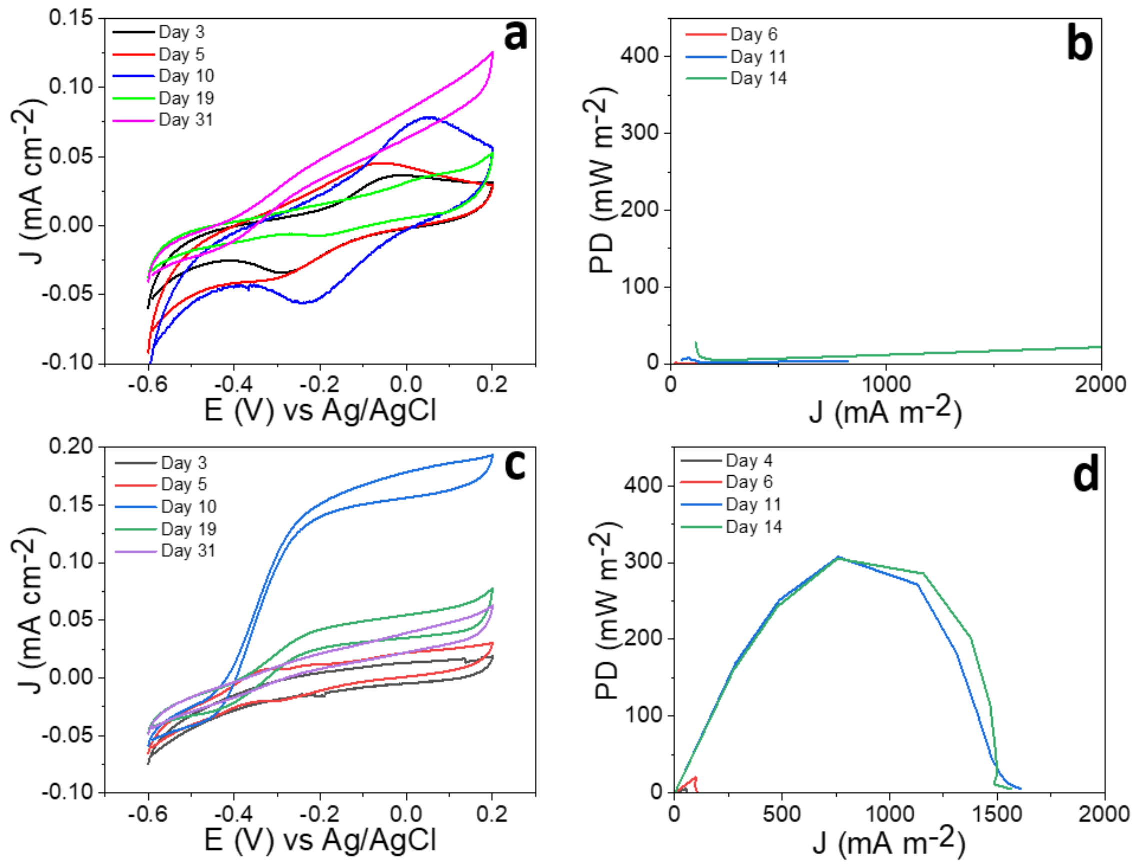

Figure S5. In situ CV curves of bare graphite anodes in the anodic compartment of MFC devices obtained at 5 mV s^{-1} after day 3, day 5, day 10, day 19 and day 31 and their corresponding power density curves obtained at day 4, 6, 11 and 14; GR_bare#1 (a, b) and GR_bare#3 (c,d).

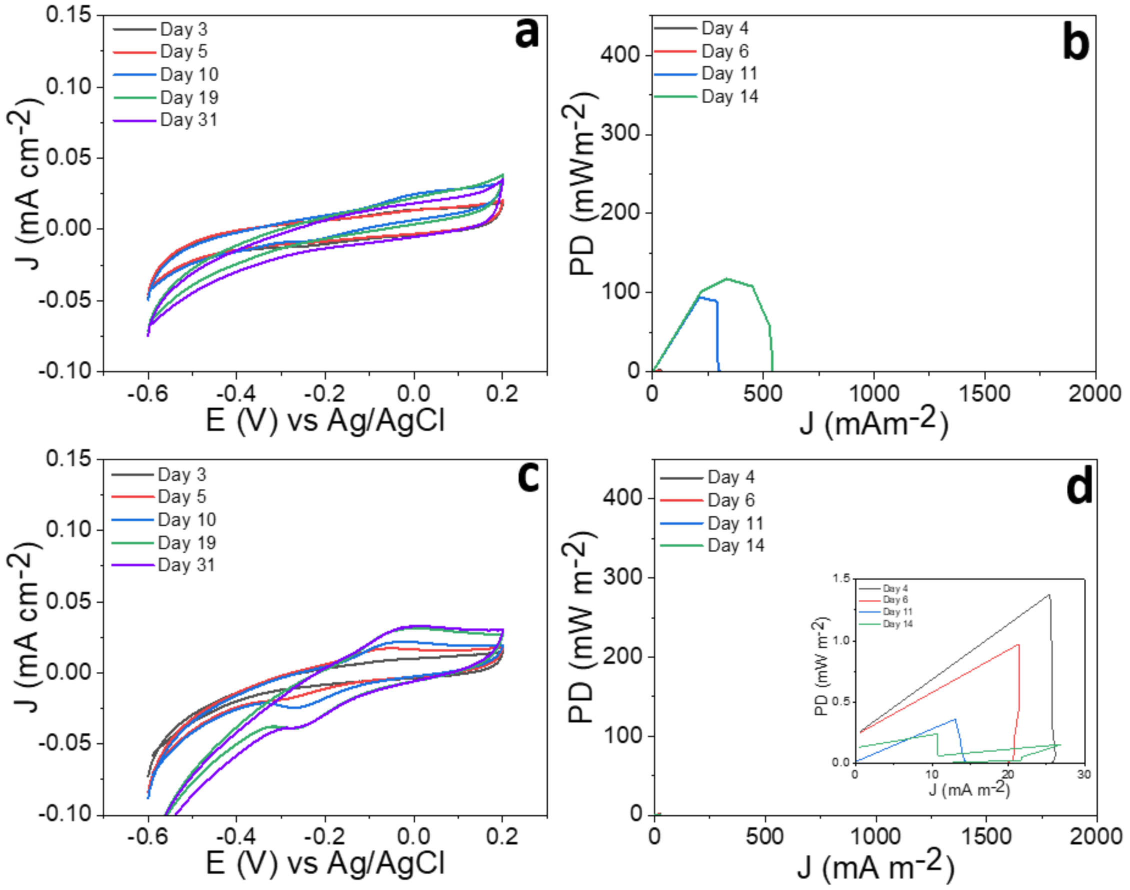

Figure S6. In situ CV curves of GR_Lac anodes in MFC devices obtained at 5 mV s^{-1} after day 3, day 5, day 10, day 19 and day 31 and their corresponding power density curves obtained at day 4, 6, 11 and 14; GR_Lac#1 (a, b) and GR_Lac#2 (c,d).

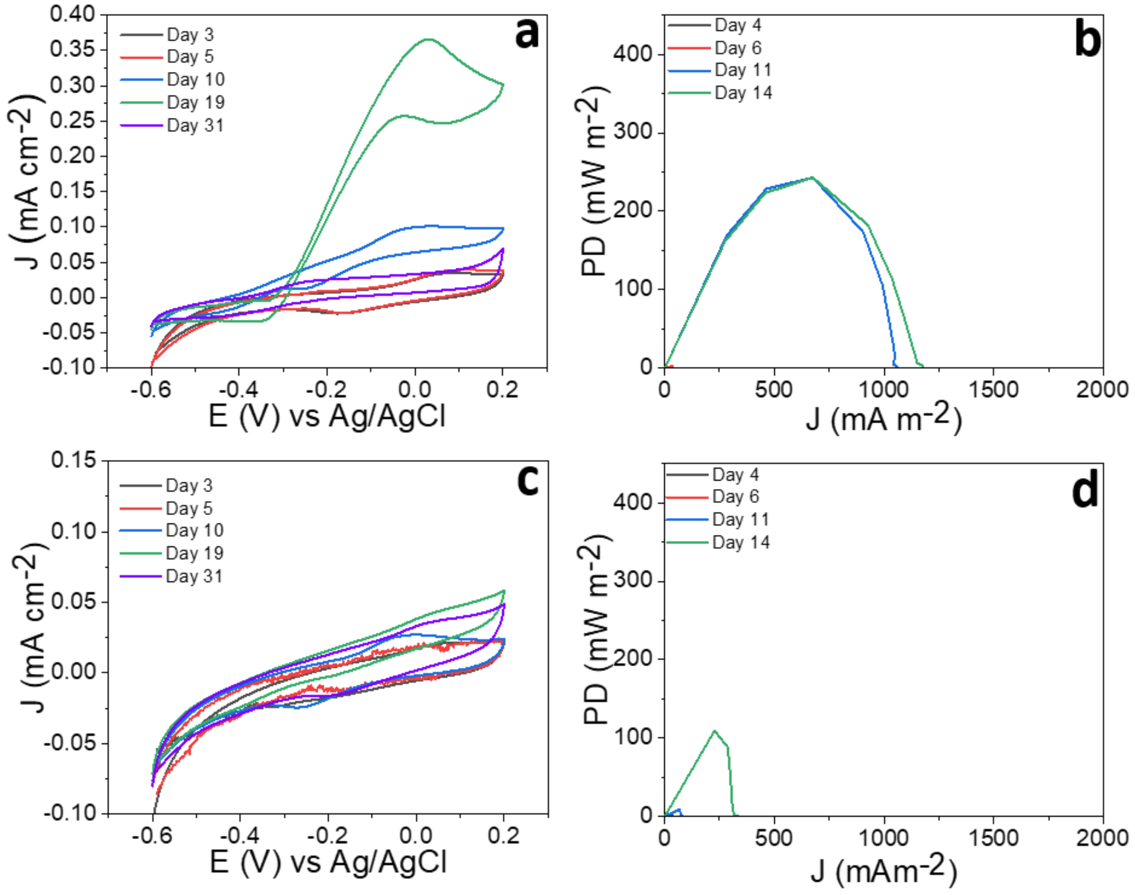

Figure S7. In situ CV curves of GR_Man anodes in MFC devices obtained at 5 mV s^{-1} after day 3, day 5, day 10, day 19 and day 31 and their corresponding power density curves obtained at day 4, 6, 11 and 14; GR_Man#1 (a, b) and GR_Man#2 (c,d).
